# Rhomboid intercostal block versus erector spinae plane block for perioperative analgesia in patients undergoing reduction mammoplasty: a prospective randomized study

**DOI:** 10.1186/s12871-025-03567-0

**Published:** 2026-02-06

**Authors:** Shereen E. Abd Ellatif, Rehab A. Wahdan, Rasha A. Wahdan, Emad Salah Ibrahim, Asmaa M. Galal Eldin

**Affiliations:** 1 Department of Anesthesia, Intensive Care, and Pain Management, Faculty of Medicine, Zagazig University, Al- qwmiah, Zagazig, Egypt; 2 Department of Plastic and Reconstructive Surgery, Faculty of Medicine, Zagazig University, Zagazig, Egypt

**Keywords:** Analgesia, Erector spinae plane block, Fascial plane blocks, Plastic surgery, Reduction mammoplasty, Regional anesthesia, Rhomboid intercostal block

## Abstract

**Background:**

Erector spinae plane block (ESP) is inevitably effective in posterior thoracic surgeries, but its efficacy and safety in anterior thoracic surgeries—particularly bilateral surgeries—remain in dispute. This study aimed to evaluate the analgesic efficacy of rhomboid intercostal block (RIB) and ESP after reduction mammoplasty.

**Methods:**

This prospective randomized controlled study was conducted on 72 patients scheduled for reduction mammoplasty. Patients were randomly assigned to three groups. Group C received general anesthesia, and groups ESP and RIB received ESP and RIB blocks, and general anesthesia. The primary outcome was the first rescue analgesic time. Postoperative pain score, 24 h nalbuphine consumption, and dermatomal level were the secondary outcomes.

**Results:**

Compared with those in the ESP and control groups, the first rescue analgesic time and 24 h nalbuphine consumption were significantly longer and lower, respectively, in the RIB group. Furthermore, the RIB group had the lowest significant pain scores within the first 6 h postoperatively. Significant sensory blocking of the anterior hemithorax from T2-T9 was accomplished by the RIB, while more dermatomal blockade of the posterior hemithorax from T2-T9 was provided by the ESP.

**Conclusions:**

RIB is more effective than ESP in managing pain after reduction mammoplasty. It prolongs the duration of analgesia, reduces postoperative nalbuphine consumption and is associated with a lower incidence of complications; hence, RIB can be utilized as a promising alternative in anterior thoracic wall surgeries such as reduction mammoplasty.

**Trial registration:**

This clinical trial was approved by Zagazig University’s Institutional Review Board (IRB) (ZU-IRB# 11408/January15, 2024) and ClinicalTrials.gov (NCT06225895, registration date January 26, 2024), with the first research participant enrolled on February 1, 2024.

## Introduction

Reduction mammoplasty is the gold standard procedure for symptomatic breast hypertrophy, and it is also used for contralateral breast symmetrization following breast cancer surgery [1]. Symptomatic hypermastia affects the quality of life of millions of women worldwide. The most frequent symptoms shown by more than two-thirds of patients are shoulder grooving and back, shoulder, and neck pain [2]. Reduction mammoplasty has proven to be an effective treatment, both aesthetically and functionally, with consistently high patient satisfaction [3, 4].

Optimal pain management is an essential component of enhanced recovery after surgery protocols that are becoming the standard of care because they have been shown to reduce postoperative complications and expedite recovery [5, 6]. However, postoperative pain is still inadequately managed. Opioids remain the mainstay of perioperative pain management, despite well-recognized adverse events, including nausea, vomiting, pruritus and respiratory depression [6, 7].

Regional anesthesia is believed to be one of the formats for effective perioperative pain control. Plane blocks such as the paravertebral block (PVB), serratus anterior plane (SAP) block, pectoral nerve block, and erector spinae plane block have gained popularity during multimodal analgesia after breast surgery [8–10].

Erector spinae plane block (ESP) was initially introduced by Forero et al. in 2016 [11] and offers extensive analgesia in thoracic surgery. It can be used as a substitute for paravertebral block (PVB) because it is less intrusive, simpler, and safer to apply plane blocks that are applied in the plane of the spine’s erector muscles [12, 13].

Rhomboid intercostal block (RIB) was described in 2016 as an alternative to thoracic epidural analgesia [14]. The local anesthetic agent is delivered into the plane between the rhomboid major and intercostal muscles. According to earlier cadaveric research, the dye is distributed widely in the craniocaudal and anteroposterior regions, which may explain why the RIB can provide effective analgesia covering the anterior and posterior hemithorax [15]. RIB has demonstrated efficacy in both breast and thoracoscopic procedures [16–18]. Moreover, the injection site for RIB is more peripheral than that for ESP thus the lateral branches of the intercostal nerves receive most of the local anesthetic instead of the paravertebral and epidural region with their unwanted consequences [19]. Therefore, we hypothesize that bilateral RIB might be more effective and safer in controlling pain after anterior thoracic surgeries. Thus, this study aimed to evaluate and compare the impact of RIB and ESP on prolonging analgesic effect and reducing opioid consumption after reduction mammoplasty.

## Patients and methods

### Study population

The Zagazig University Hospitals hosted this prospective randomized controlled clinical trial from February to September 2024. Following approval by the institutional review board (The Research Ethical Committee of the Faculty of Medicine, Zagazig University) with reference number ZU-IRB#: 11,408//15/1/2024, the study was registered with ClinicalTrials.gov (NCT06225895). The study followed the ethical guidelines of the Declaration of Helsinki. This randomized controlled trial was developed and carried out in accordance with the Consolidated Standards of Reporting Trials (CONSORT) (http://www.consort-statement.org).

Adult female patients aged 18–65 years with a BMI ≤ 35 kg/m^2^, ASA I or II and scheduled for elective bilateral reduction mammoplasty under general anesthesia were included in this study. Pregnant females or patients with a history of bleeding disorders, psychiatric disorders, communication difficulties (e.g., cognitive impairment, language barriers, or hearing loss), sepsis, compromised renal or hepatic functions, allergy to the LA agent used, or skin lesions at the needle insertion site were excluded from the study.

- Randomization

Patients were recruited prior to their admission to the preoperative anesthesia clinic. The study’s details were disclosed prior to providing signed informed consent.

A random number table was generated via Excel software. The sequence was kept in sealed opaque envelopes by a doctor who was not engaged in patient assessment. An independent anesthetist who was not involved in any additional research procedures opened these envelopes. Throughout the study, the surgeon, anesthesiologists, data collectors, and statisticians remained blind to the group allocations, while the patients and the investigator who performed the blocks were not blinded due to the nature of the interventions. However, the investigator was not involved in any further study steps.

Seventy-two patients were randomly assigned to three equal groups (24 each): the C group (control group) underwent surgery under general anesthesia; the ESP group received bilateral ESP block with 40 ml of 0.25% bupivacaine and general anesthesia; and the RIB group received bilateral RIB with 40 ml of 0.25% bupivacaine and general anesthesia.

### Preoperative assessment

Preoperative visits were made the night before surgery to discuss the study protocols, assess the patient’s medical status, exclude drug sensitivity, and explain the anesthetic regimen and fasting schedule (6 h for solids and 2 h for fluids).

On physical examination, vital signs and chest and cardiac conditions were documented to exclude contraindications. All patients were subjected to a complete blood count, random blood sugar, coagulation profile, hepatitis marker, liver function and kidney function tests.

On a visual analogue scale (VAS) score [20] of 0–10, the patient was asked to quantify postoperative pain, where 0 = no pain and 10 = maximum worst pain, to record the postoperative pain level. 

### Intraoperative

Standard monitors, such as ECG, pulse oximetry, noninvasive blood pressure monitoring, and capnography, were attached to the patients, and baseline data were recorded. An IV (intravenous line) was inserted. The surgeon placed markings of his planned surgery on both breasts and the anterior chest wall. Prior to block performance, 3–5 mg of midazolam was given to the patient. The same anesthesiologist conducted all block procedures on both groups of patients.

**Fig. 1:**
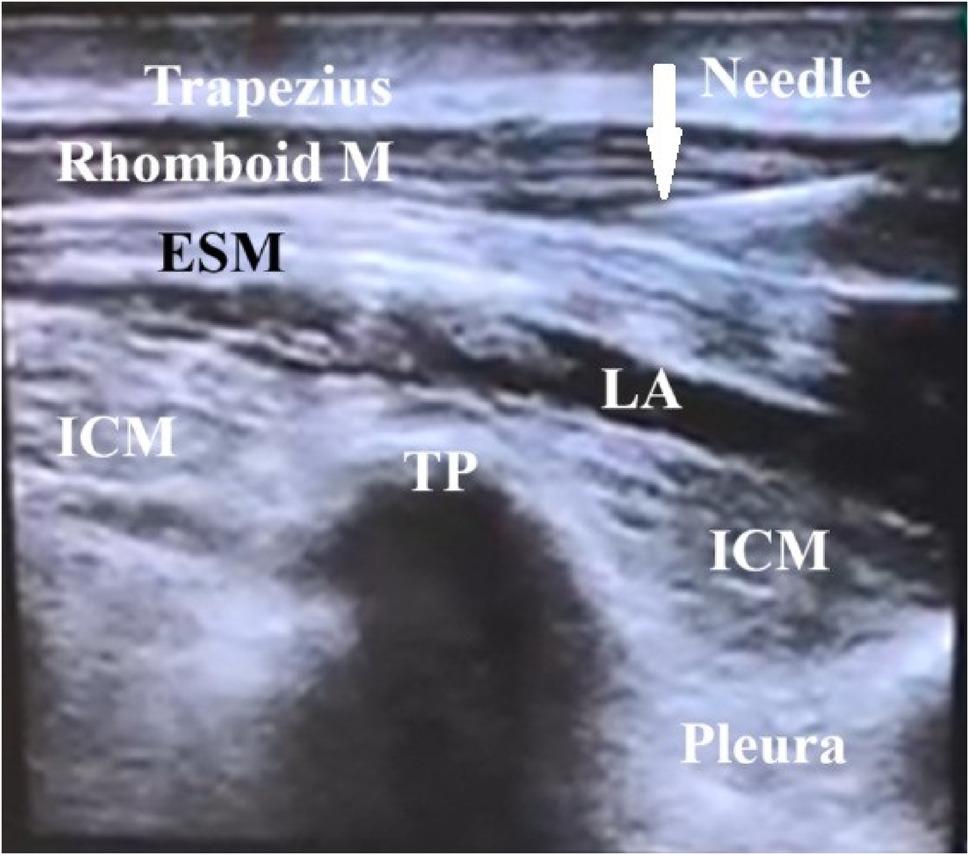
Sonographic image of the Erector spinae plane block. ESM: Erector spinae muscle, ICM: intercostal muscle, TP: transverse process LA: local anesthetic spread between the ESM and ICM, white arrow shows needle trajectory

- ❖ *ESP block technique*:

The patient was kept in the lateral decubitus position. Following sterilization and draping of the skin, a high-frequency ultrasound probe (Siemens Medical Solutions, Inc., Mountain View, CA 94043, USA) was positioned longitudinally at the level of the T5 spinous process and 3 cm laterally from the midline on one side, revealing the erector spinae muscle lying beneath the trapezius and rhomboid major muscles [21]. In-plane insertion of an 80 mm 22-gauge block needle (Stimuplex D, B-Braun, Germany) was performed from cranial to caudal until the tip contacted the T5 transverse process. Following hydrodissection with 1 ml of normal saline, 20 ml of 0.25% bupivacaine was injected deep into the erector spinae muscle (Fig. 1). The contralateral side received 20 ml of 0.25% bupivacaine, and the same process was repeated. 

**Fig. 2:**
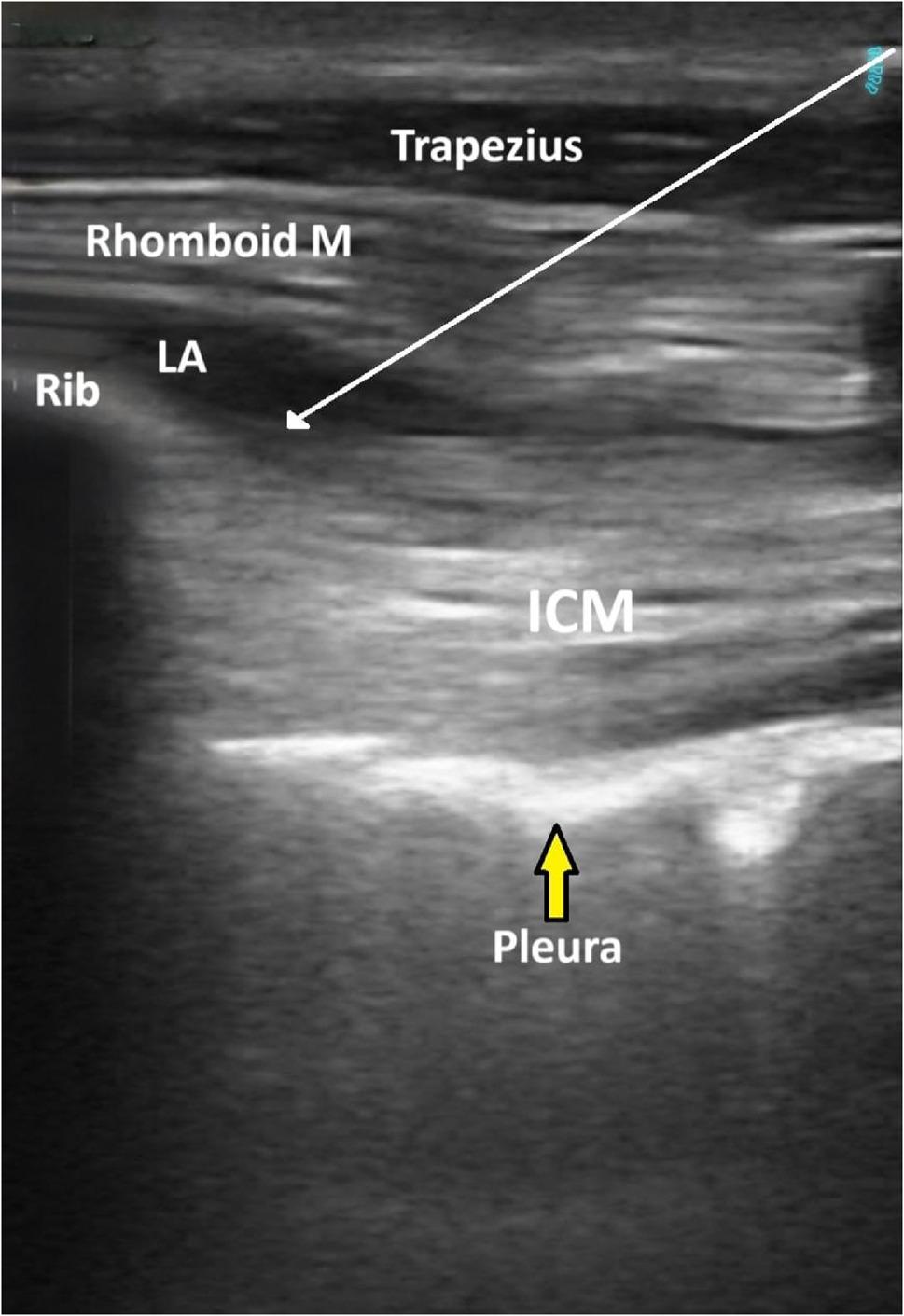
Sonographic image of the Rhomboid intercostal block. Rhomboid M: Rhomboid major muscle, ICM: intercostal muscle, LA: local anesthetic spread between the Rhomboid M and ICM, white arrow shows needle trajectory

- ❖* Technique of the RIB*:

The patient was kept in the lateral position, and the ipsilateral arm was extended to move the scapula laterally and open the space. The skin was sterilized and draped, followed by the placement of a linear probe (Siemens Medical Solutions, Inc., Mountain View, CA 94043, USA) in an oblique sagittal plane medial to the lower border of the scapula, the triangle of auscultation, at the level of the T5 to T6 vertebrae. The rhomboid major muscle is located beneath the trapezius muscle. The pleura, lungs and intercostal muscles were also identified [18]. Using an in-plane approach, a 22-gauge, 80 mm stimuplex needle was placed in the craniocaudal direction into the fascial plane between the rhomboid major and the intercostal muscles. The needle tip position was confirmed via hydrodissection with 1 ml of normal saline, after which 20 ml of 0.25% bupivacaine was injected underneath the rhomboid major muscle (Fig. 2). The contralateral side received 20 ml of 0.25% bupivacaine, and the same process was repeated.

Participants of the two interventional groups underwent sensory assessment using standardized cold sensation test with iced solutions every 5 min for 30 min after injecting LA. The data was recorded as the area of sensory loss at the midclavicular line (MCL), midaxillary line (MAL)and at scapular line (SL)compared to the ipsilateral shoulder’s intact sensibility. The same process was repeated on the contralateral side. A block was considered successful if at least three dermatomes from T3–T6 at MAL and MAL, were covered bilaterally; otherwise, a failed block was declared after 30 min of LA injection, and this case was withdrawn from the study. The onset time of the block (from injecting LA until achieving the desired successful sensory level) and its maximum dermatomal level were recorded. An assistant who was not involved in the block performance or the following study steps conducted a block assessment.

General anesthesia was standardized for all patients. Preoxygenation with 100% O2 was applied for three to five minutes before the induction of anesthesia. Anesthesia was induced with 2 mg/kg propofol and 1–2 mcg/kg fentanyl, followed by 0.15 mg/kg cisatracurium to facilitate endotracheal intubation with a suitable endotracheal tube size. The lungs were ventilated in volume-controlled mode to maintain normocapnia (ETCO_2_ = 35–45 mmHg), and an isoflurane/O2 mixture was administered. The concentration of the inhalational agent will be 1.2 MAC of isoflurane. Heart rate (HR) and mean arterial blood pressure (MAP) were measured every 5 min until the end of surgery. Incremental doses of fentanyl were given if inadequate analgesia was detected (increased HR or MAP > 20% from baseline values) after exclusion of other causes. The total dose of fentanyl consumed intraoperatively was recorded.

- ❖* Surgical technique*:

The surgical procedure was done by the same plastic surgeon with over 10 years’ experience. All patients underwent a standardized surgical technique using an inferior pedicle-wise pattern reduction mammoplasty.

After completion of the surgery, inhalational anesthesia was stopped, and the patient received neostigmine (0.05 mg/kg) and atropine sulfate (0.01 mg/kg) to reverse residual neuromuscular block. All patients received 1 gm of paracetamol as standard intravenous infusion analgesia. The patient was transferred to the postoperative recovery unit (PACU) after smooth extubation.

According to a predetermined schedule, all patients in the surgical ward received 200 mg celecoxib orally twice daily and paracetamol 1 gm/8 hours (maximum dose: 4 g/day) as standard postoperative analgesics. Rescue analgesia in the form of 0.1 mg/kg IV of nalbuphine was injected if the patient reported VAS score of ≥ 3 or was asked for more analgesia according to the VAS score during the first 24 h after surgery (the maximum single dose was 20 mg, and the maximum daily dose was 160 mg).

### Parameter evaluation

- Patient characteristics: Age, BMI, and ASA physical status.
- The 1st rescue analgesic time (primary outcome) was the time it took to request the first postoperative analgesic (nalbuphine) from the end of surgery until the patient reported a VAS score of ≥ 3.

 • Secondary outcomes:
  - ➢ The total dose of nalbuphine, a rescue analgesic, was taken during the first 24 hours after surgery.
  - ➢ The technique time (min) was defined as the time between placing the ultrasound probe on the patient's skin and injecting the LA.
  - ➢ The visual analogue scale (VAS) score was assessed at rest and during movement (arm movement of 90° and sitting from the lying down position) at PACU, 1 h, 3 h, 6 h, 12 h, and 24 h postoperatively.
  - ➢ The number of covered dermatomes was counted after 30 minutes of finishing the block via cold loss sensation with iced solutions.
  - ➢ Heart rate (HR) and mean arterial blood pressure (MAP) were recorded at baseline before the fascial plane blocks and then immediately and 15, 30, 60, 120, 180, and 240 min after skin incision and at the end of surgery.
  - ➢ Intraoperative cumulative fentanyl (µg).
  - ➢ Operative time (min).
  - ➢ The perioperative complications included opioid complications such as respiratory depression, nausea, vomiting, bradycardia, and hypotension and block complications such as needle injury to essential organs and local anesthetic systemic toxicity [LAST].
  - ➢ Patient satisfaction was assessed 24 h postoperatively via a satisfaction score (1‑not satisfied, 2‑fairly satisfied, 3‑satisfied, or 4‑extremely satisfied) [22].

### Sample size calculation

The time to first patient-controlled analgesia (PCA) request was 70.4 ± 60.9 min and 29 ± 43.6 min for patients receiving erector spinae plane block (ESP) and sham groups, respectively [23]. The sample size was calculated to be 66 allocated into three groups, and 10% was added for drop out, so the total sample size was 72 cases (24 in each group) at a confidence level of 95%, and the power of the study was 80%.

### Statistical analysis

The statistical package for social sciences, SPSS, version 28, was used to analyze the data. The Shapiro‒Wilk test was used to confirm the assumptions for use in the parametric tests. Categorical variables are presented as absolute frequencies and were compared via the Chi Square test and the Monte Carlo test when applicable. The chi-square trend test was used to compare ordinal data between two groups. Quantitative variables are presented as the means and standard deviations or medians and interquartile ranges (IQR) according to the type of data. Independent sample t tests (for normally distributed data) were used to compare quantitative data between two groups. One-way ANOVA was used for data that were normally distributed, and the Kruskal‒Wallis test was used for data that were not normally distributed to compare quantitative data between more than two groups. Bonferroni and pairwise comparisons were used to identify differences between each of the two groups when the difference was significant. The value of *P* < 0.05 was set for statistical significance. If *p* ≤ 0.001, a highly significant difference was detected.

## Results

**Fig. 3:**
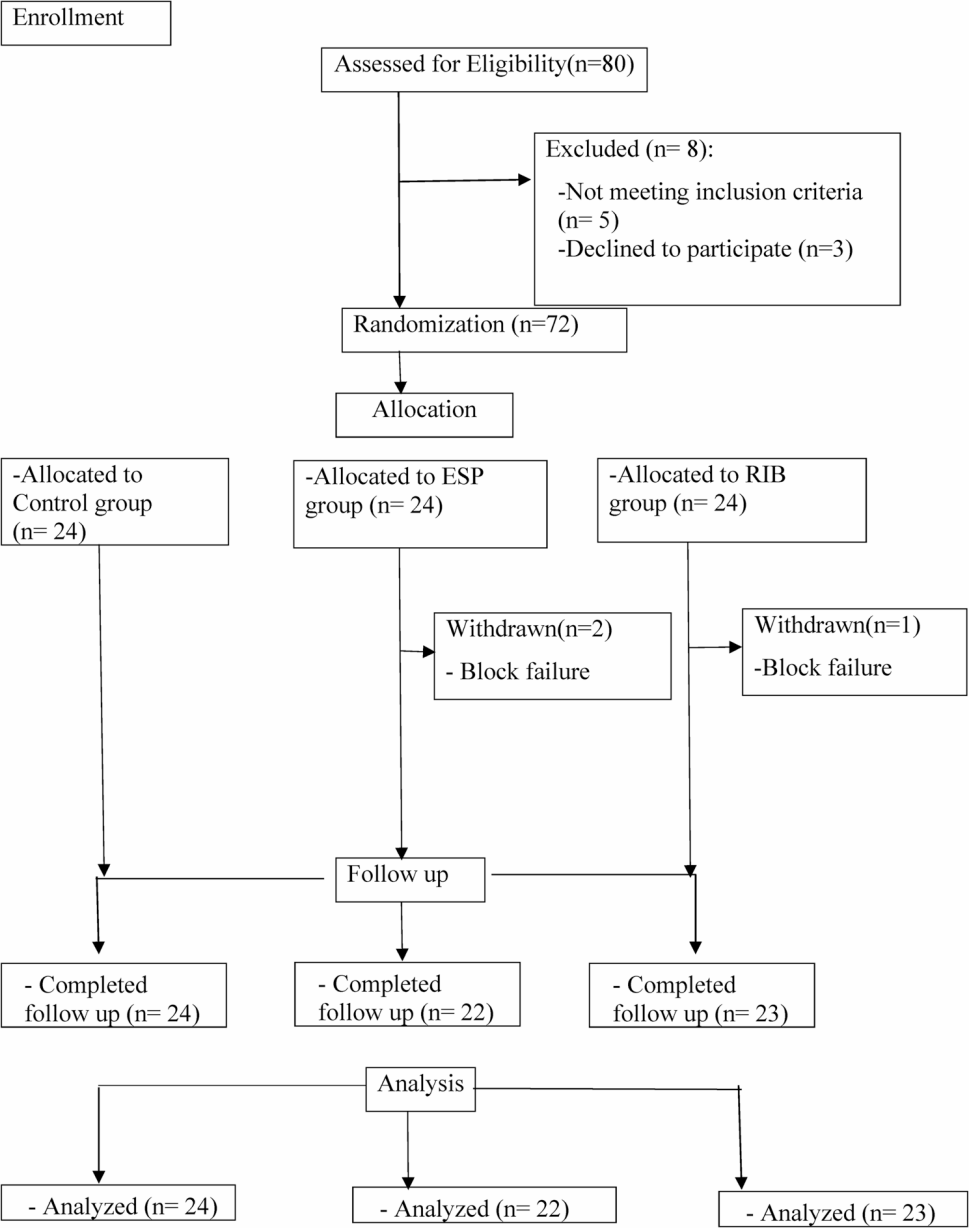
CONSORT flow diagram

Eighty patients whose eligibility was assessed in this study were scheduled for bilateral reduction mammoplasty under general anesthesia. The CONSORT flow diagram revealed that eight patients were excluded: three patients chose not to participate, and five patients met one or more of the exclusion criteria. Seventy-two patients were thus randomly divided into three equal groups of 24 patients each. After that, two patients in the ESP group and one patient in the RIB group were withdrawn from the study due to failure of the blocks to achieve the target dermatomal level necessary for adequate postoperative analgesia (Fig. 3).

There was a statistically nonsignificant difference between the studied groups in terms of age, BMI, ASA score and operative time. However, there was a statistically significant increase in total intraoperative fentanyl consumption in the control groups compared with the ESP and RIB groups, with no difference between the latter two groups. For the block data, there was no significant difference between the ESP and RIB groups in terms of the time of block performance or onset of the sensory block. (Table 1).

**Table 1:** Patient characteristics and operative data

**Characteristics**	**Control group** **(n=24)**	**ESP group** **(n=22)**	**RIB group** **(n=23)**	**P value**	**Bonferroni**
Age (yrs):					
Mean ± SD	37.17 ± 11.76	39.95 ± 10.82	37.74 ± 11.5	^a^ 0.686	
BMI (kg/m2):					
Mean ± SD	27.42 ± 2.98	27.36 ± 3.0	27.91 ± 2.58	^a^ 0.774	
ASA: N (%)					
I:	15 (62.5%)	15 (68.2%)	16 (65.2%)	^b^ 0.922	
II:	9 (37.5%)	7 (31.8%)	7 (34.8%)		
Block performance time(min):					
Mean ± SD	**------**	22.86 ± 3.98	21.83 ± 4.21	^c^ 0.401	
Sensory block onset (min):					
Mean ± SD	**------**	21.18 ± 3.58	20.78 ± 3.09	^c^ 0.69	
Total intraoperative fentanyl (µg):					
Mean ± SD	328.33 ± 35.17	220.0 ± 31.62	206.96± 28.19	^**a**^ <0.001**	**P**^**1**^<0.001** P^**2**^<0.001** P^3^ = 0.531
Operative time:					
(min) Mean± SD	257.08 ± 31.41	256.36 ± 27.87	260.43 ± 27.88	^a^ 0.881	

Data are expressed as the means ± SDs, numbers and percentages

ESP Erector spinae plane block, RIB Rhomboid intercostal block, BMI Body mass index

^a^ANOVA test

^b^chi-square test

^c^independent sample t test

n = total number of patients in each group

P^1^: Control group versus ESP group P2: Control group versus RIB group. P3: ESP group versus RIB group

P >0.05 indicated a nonsignificant difference

**p≤0.001 is highly statistically significant

Regarding the first time of rescue analgesia and total nalbuphine consumption in the first 24 h postoperatively, statistically significant differences were noted among the three studied groups, where the longest time passed and the lowest consumption, respectively, was in the RIB group, followed by the ESP group; meanwhile, the shortest duration and highest consumption were recorded in the control group (Table 2).

**Table 2:** Postoperative analgesic data among the studied groups

**Variables**	**Control group (n=24)**	**ESP group** **(n=22)**	**RIB group** **(n=23)**	**P value**	**Bonferroni**
Time of first rescue					
analgesia (min):					
Mean ± SD	60.13 ± 8.34	283.23 ± 33.64	308.04 ± 37.26	^**a**^ <0.001**	**P** ^**1**^ <0.001** **P** ^**2**^ <0.001** P ^**3**^ =0.017*
Nalbuphine					
Consumption (mg) in 24 h.:					
Mean ± SD	23.38 ± 3.68	13.05 ± 2.36	10.57 ± 2.11	^**a **^ <0.001**	**P** ^**1**^ <0.001** **P** ^**2**^ <0.001** **P** ^**3**^ =0.013*

The data are expressed as the means ± SDs

*ESP* Erector spinae plane block, *RIB* Rhomboid intercostal block

^a^ANOVA test

n = total number of patients in each group

P^1^: Control group versus ESP group

P^2^: Control group versus RIB group

P^3^: ESP group versus RIB group

*P ≤0.05 indicated a significant difference, and **p ≤0.001 was highly significant

**Fig. 4:**
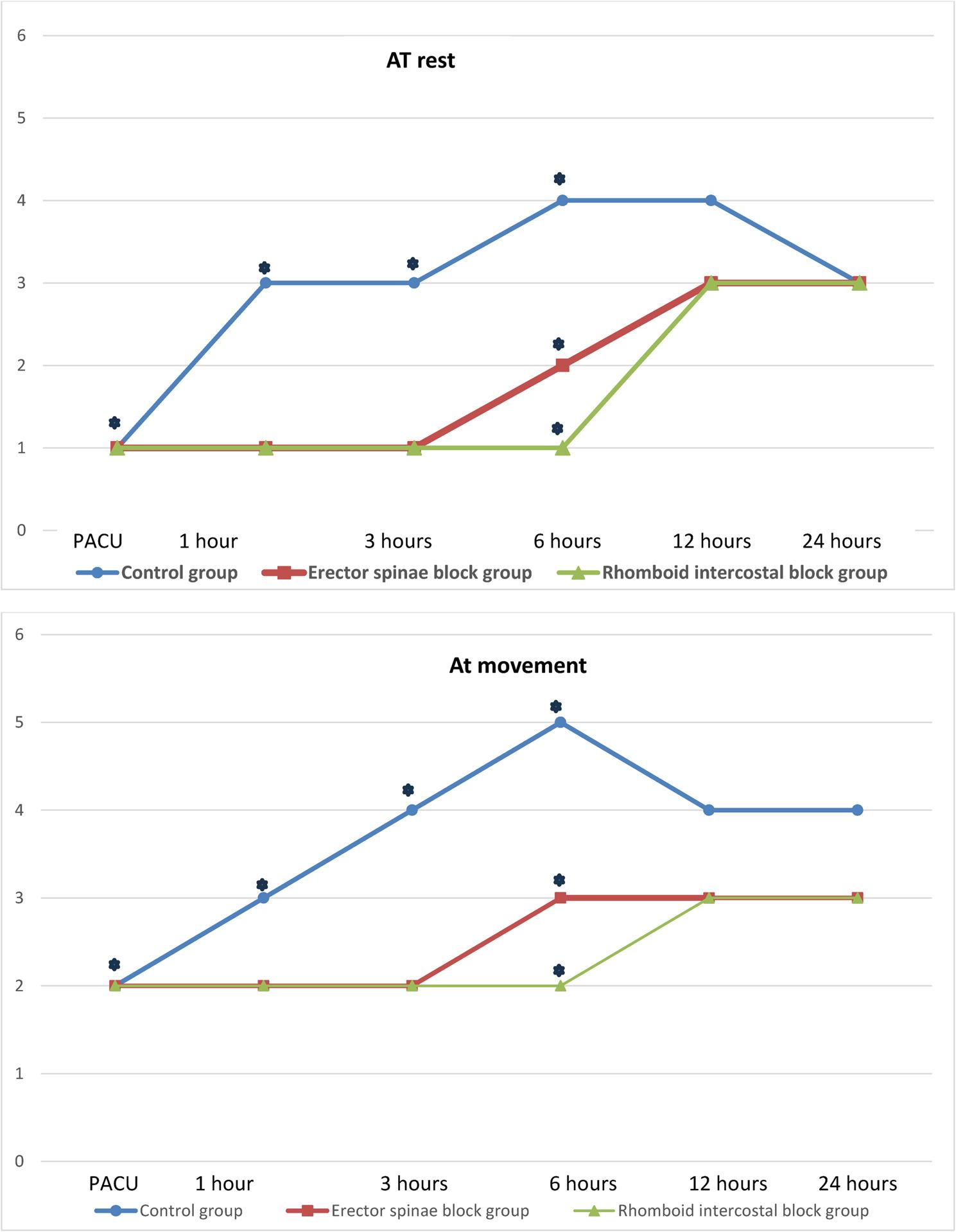
Median postoperative visual analog scale (VAS) score at rest and with movement among the studied groups. Kruskl Wallis test

The VAS scores at rest(r) and during movement(m) were significantly increased in the control group compared to the ESP and RIB groups (with no difference between the last two groups) at PACU; median (IQR) ((r 1(1–2), m 2(2–3)), ((r 1(0–1), m 2(1–2)), (r 1(0–1), m 2(0–2)) *p* ≤ 0.001, respectively, and at 1 and 3 h postoperatively. At 6 h postoperatively at rest and with movement, the RIB group presented the lowest significant VAS score among the three study groups (for pairwise comparisons, the difference was significant between each of the two individual groups). However, at 12 and 24 h postoperatively, no significant difference was found among the three studied groups at rest or with movement, indicating that the two intervention groups still had lower scores (Fig. 4).

**Fig. 5:**
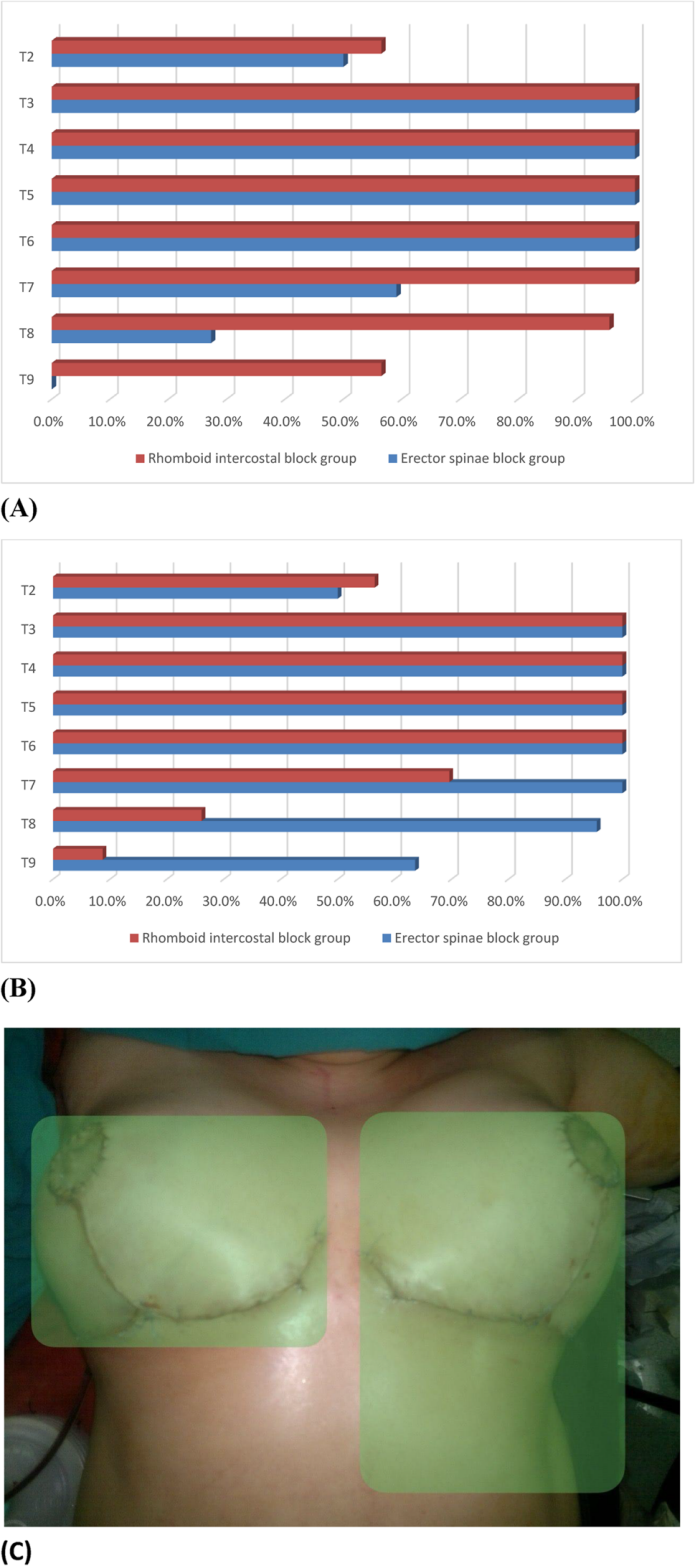
**A**. Sensory block distribution of the anterolateral hemithorax, **B**. Sensory block distribution of the posterior hemithorax in the two block groups. χ2 chi-square test for trend. **C**. Anterior thoracic distribution of sensorial blockage area of more than 90% of female patients shown with a green shadow for ESP group on the right side and for RIB group on the left side of the image

A statistically significant difference was observed in the percentage of sensory block distribution between the ESP and RIB groups. The RIB block achieved more extensive dermatomal coverage of the anterior hemithorax, whereas the ESP provided greater dermatomal coverage in the posterior hemithorax (Fig. 5A, B, C).

**Fig. 6:**
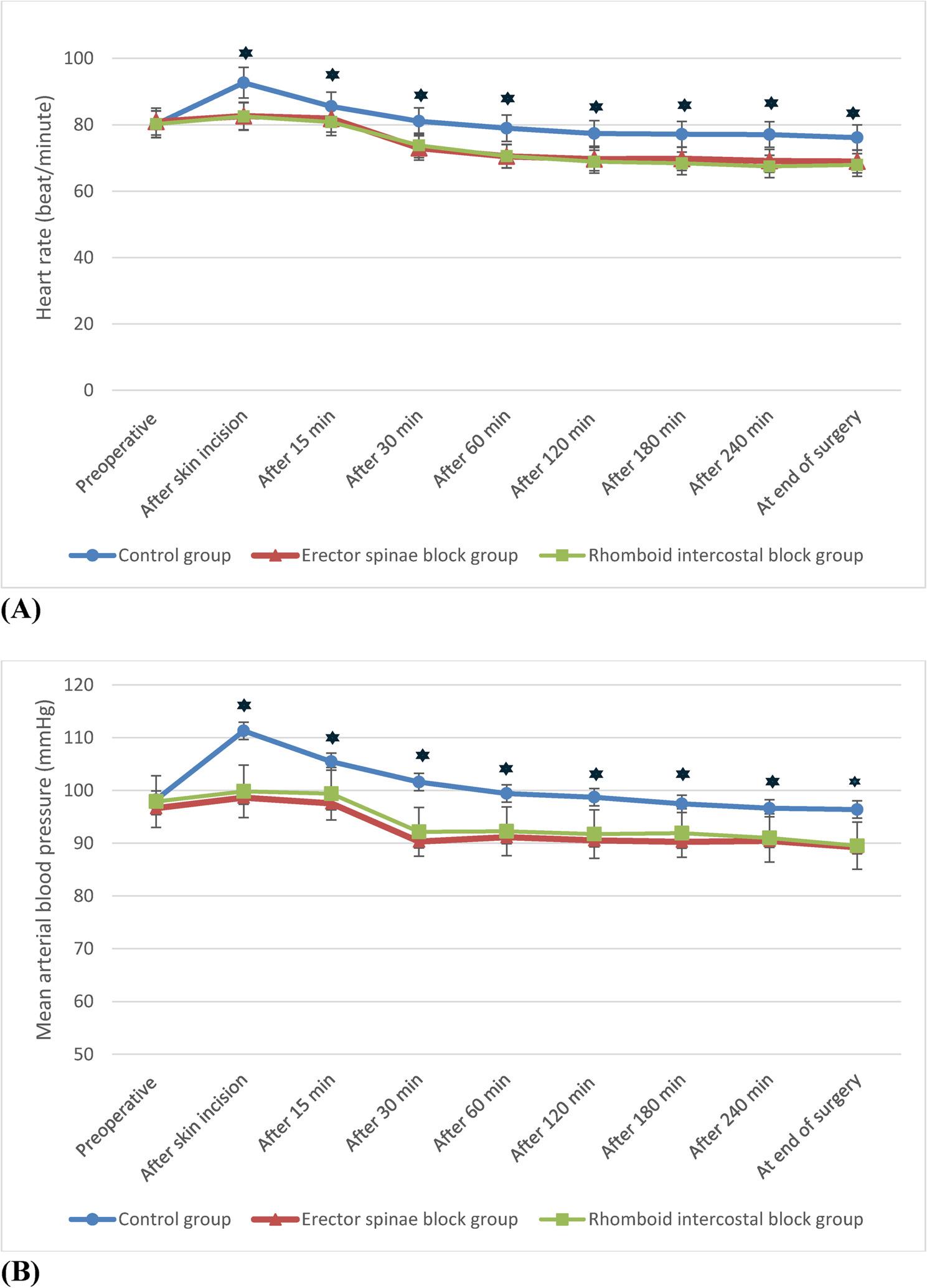
Intraoperative mean heart rate (**A**) and arterial blood pressure (**B**) in the studied groups. One-way ANOVA

There was no significant difference among the studied groups regarding hemodynamics (HR and MAP) at baseline. However, HR and MAP were significantly greater in the control group than in the ESP and RIB groups at all measuring times from the time of skin incision until the end of surgery, with no significant difference detected between the two intervention groups (Fig. 6).

A statistically significant increase in the incidence of nausea and vomiting was detected in the control group compared with the other two groups; however, no significant difference was found between the three groups with respect to other opioid-related complications or block-related complications. Patients in the ESP and RIB groups were more satisfied (with no significant difference between the two groups) than were those in the control group (Table 3).

**Table 3:** Postoperative complications and patient satisfaction among the studied groups

**Variables**	**Control group** **(n=24)**	**ESP group** **(n=22)**	**RIB group** **(n=23)**	**P value**	**Chi square for trend test**
Nausea & vomiting N (%):					
No:	16 (66.7%)	20 (95.5%)	22 (95.7%)	^**a **^ 0.014*	**P**^**1**^ = 0.046* P^**2**^= 0.004* P^3^ = 0.488
Yes:	8 (33.3%)	2 (4.5%)	1 (4.3%)		
Bradycardia N (%)					
No	22 (91.7%)	22 (100%)	22 (95.7%)	^a ^0.812	
Yes	2 (8.3%)	0 (0%)	1 (4.3%)		
Hypotension N (%)					
No	22 (91.7%)	19 (86.4%)	23 (100%)	^a^0.233	
Yes	2 (8.3%)	3 (13.6%)	0 (0%)		
Respiratory depression N (%)					
No	24 (100%)	22 (100%)	23 (100%)		
LA toxicity N (%):					
No:	-----	22 (100%)	23 (100%)		
Needle trauma N(%)					
No:	-----	22 (100%)	23 (100%)		
Patient Satisfaction					
-Not satisfied	14 (58.3%)	2 (9.1%)	2 (8.7%)		
-Fairly dissatisfied	7 (29.2%)	6 (27.3%)	5 (21.7%)	^**a **^ <0.001**	**P**^**1**^<0.001** P^**2**^ <0.001** P^3^ = 0.333
-Satisfied	2 (8.3%)	10 (45.5%)	7 (39.1%)		
-Extremely satisfied	1 (4.2%)	4 (18.2%)	9 (39.1%)		

Data are expressed as numbers and percentages

ESP Erector spinae plane block, RIB Rhomboid intercostal block, LA Local anesthesia

^a^Monte Carlo test

n = total number of patients in each group

P^1^: Control group versus ESP group

P^2^: Control group versus RIB group

P^3^: ESP group versus RIB group

*P* >0.05 indicated a nonsignificant difference, ^*^P ≤0.05 indicated a significant difference, and ^**^p≤0.001 was highly significant

## Discussion

The present study evaluated the analgesic effect of bilateral ultrasound-guided RIB and ESP for reduction mammoplasty and provided compelling evidence that RIB offers significant benefits in terms of perioperative analgesia; the RIB group had a longer first-rescue analgesic time and less nalbuphine consumption within the first 24 h postoperatively than did the ESP and control groups. Moreover, the RIB and ESP groups had lower VAS scores at rest and during movement in the PACU and in the first three postoperative hours than did the control group; however, at 6 h postoperatively, the RIB group recorded the lowest significant VAS scores among the three study groups. Furthermore, no differences were found among the study groups regarding opioid-related complications or block-related complications. This discussion explores the latent reasons and potential mechanisms behind these results, integrating the findings with literature to provide a comprehensive understanding of the impact of RIB.

Despite being referred to as a “fascial plane block,” the exact mechanism of action of the ESP block is still elusive, and its clinical effect is unpredictable. Hypothesized explanatory mechanisms of the LA spread derived from cadaveric and imaging models indicated reliable coverage of the dorsal rami; extension toward the ventral rami and paravertebral space is frequently noted, but this is not replicable [24, 25]; hence, its clinical efficacy is clear in providing reliable analgesic coverage from the back to the axillary midline; however, its analgesic coverage to the anterior chest wall is still debatable because it frequently extends to many, but not all, patients [26, 27].

Another point of view was presented by Choi et al., who assumed that the ESP block might be volume dependent and result in extensive spread. Nevertheless, they concluded that even increasing the volume up to 30 ml of dye increased the spread to the posterior rami of the spinal nerves and lateral cutaneous branches of the intercostal nerves more than the extent to the paravertebral space. Surprisingly, they could not find any clear intercostal nerve involvement in the intercostal space. Moreover, they noted that, in living subjects, movement of the chest during inspiration and expiration may cause a delay in the diffusion of LAs to the intercostal or paravertebral spaces [28]. Notably, Luftig et al. reported that the potential for LAST occurred when ESP blocks with more than 40 ml of diluted LAs were used [29]. This makes it necessary to pay some caution when it is used in bilateral surgeries such as bilateral reduction mammoplasty since larger volumes are needed with the possibility of its inefficiency since the anterior thoracic wall is the site of action.

On the other hand, RIB block results in an almost complete sensory block of T2-T9 dermatomes of the anterior hemithorax just medial from the midline and of the posterior hemithorax until immediately medial to the spinous processes [15]. Moreover, the RIB injection site is more peripheral than that used with ESP, and LA mostly travels to the lateral branches of the intercostal nerves and, to a lesser extent, to the dorsal rami of the thoracic spinal nerves rather than to the paravertebral and epidural spaces. Thus, the incidence of hypotension may decrease since the sympathetic chain blocking with RIB is not as profound and severe as it is with ESP [30]. This is something that cannot be overlooked or not demanded, thus outweighing the RIB, especially in our bilateral surgery, which requires two volumes of the injectate that increase the possibility of hypotension. In our study, intraoperative hypotension occurred in three patients in the ESP group and none in the RIB group. Several plausible reasons might account for this result; therefore, special attention in future studies should be given to differentiating whether the ESP block is the cause among other distracting factors.

These findings are consistent with those of Chen et al., who concluded in their meta-analysis that RIB was superior to intravenous analgesia in managing acute pain following thoracoscopic and breast surgeries. At early time points, preoperative RIB dramatically decreased 24-hour opioid consumption and pain scores. Additionally, the incidence of PONV was considerably lower with RIB, which was explained by the fact that RIB seldom causes postoperative opioid usage, has little influence on hemodynamics, and does not affect the vagus nerve. Additionally, block-related complications did not occur in any of their meta-analysis studies. Therefore, they concluded that RIB is a relatively safe blocking technique [19]. In addition, An et al. reported in their meta-analysis that regional anesthesia techniques including PVB, SAP, ESP, RIB and others can successfully alleviate postoperative acute pain and reduce postoperative cumulative morphine however, the RIB may be the optimal block for postoperative analgesia in breast cancer surgery [31].

Yayika et al. reported that bilateral RIB provides effective postoperative analgesia for reduction mammoplasty, where the VAS score of the two patients was < 3/10 until the 1st 14th hr. Postoperatively, no rescue analgesia was needed [18].

Regarding ESP, Bakeer & Abdallah revealed that it was less effective than pectoral nerve block II (PECS-II) block at controlling postoperative pain after modified radical mastectomy (MRM). Notably, they reported that PECS II block blocks most of the breast nerve supply, whereas ESP block results in variable spread of LA [32]. Meanwhile, Ciftci et al. reported that RIB had analgesic effects comparable to those of the PECS-II following breast surgery [33].

On the other hand, Çiftçi et al. revealed that the opioid use, pain scores, and the need for rescue analgesia were comparable between RIB and ESP for breast conserving surgery [34]. In addition, Jiang et al. and Zhang et al. reported that the first rescue analgesic time was longer, and the opioid consumption and pain score were lower in RIB and ESP than in SAP for 24 h after modified radical mastectomy (MRM) and video-assisted thoracic surgery (VATS), respectively [35, 36].

In addition, to evaluate the intraoperative analgesic effect of the two block groups, the hemodynamic values were comparable between the RIB and ESP groups at all intraoperative measurement times compared with those of the control group; moreover, intraoperative fentanyl consumption was not significantly different between the two block groups.

Among the outcomes that were evaluated in this study that could refute what was suggested before is the dermatomal coverage of each block where the RIB block achieved more statistically significant extensive sensory coverage of the anterior hemithorax from T2-T9, which qualifies it to be effective for anterolateral hemithorax surgeries such as reduction mammoplasty, whereas the ESP provided more sensory blockade of the posterior hemithorax from T2-T9, which enhances its efficiency in some surgeries, such as spine surgeries. Therefore, the preference of one block over the other depends on the surgical site.

This finding is in accordance with that of Yayik et al., who reported in their case study that bilateral RIB resulted in dermatomal blockade between T2-T7 of the anterior, lateral and posterior hemithorax and provided effective analgesia after reduction mammoplasty [18]. Moreover, Elsharkawy et al. and Abd Ellatif et al. reported that dermatomal coverage of the RIB in their studies was from T2-T9 of the anterior and posterior hemithorax and was effective for relieving the pain associated with rib fracture or breast conserving surgery, respectively [15, 37]. Similarly, Tulgar et al. reported that the ultrasonographic spread of LAs occurred from the second to the seventh rib under the rhomboid muscle in patients undergoing MRM [38]. On the other hand, Zhang et al. reported that an ESP with 20 mL of 0. The use of 5% ropivacaine caused a general loss of sensation in the posterior thorax from T3-T12, with a concentration at T6-T9. However, the anterior chest, lateral chest, and abdomen walls were not affected [27]. Furthermore, following unilateral ESP with 30 ml of dye solution, Choi et al. reported that the majority of the injectate distributes through the back muscle or fascia layer, with a median of three stained thoracic spinal nerves at the intervertebral foramen [28]. However, according to a different cadaveric study, the ESP block extended craniocaudally over the paraspinal muscles and in the intercostal space up to nine vertebral levels [39]. Others found what is surprising is that dye can also spread to the neural foramina and epidural space [40]. Therefore, the main drawback of ESP block is its inconsistent results, and further study is needed to verify its dermatomal spread in living patients.

These factors collectively constitute the main bottlenecks in the current study for providing perioperative analgesia in reduction mammoplasty, which calls for another thought, contrary to conventional wisdom, that the ESP is an ideal choice in such cases. Meanwhile, there are other fascial plane blocks, such as the RIB, that may be more efficient and safer for anterior thoracic chest wall surgeries, especially because the two blocks are easy to implement since there was no significant difference in block performance time or block onset time between them in this study. Moreover, both blocks are far from and do not interfere with the surgical site, which is considered an advantage in clinical practice.

It’s also important to note that the patients in this study were not blinded, since the blocks were performed prior to anesthesia, thus posing challenges in achieving blinding, and we believe that it is immoral to subject patients to the risk of a sham block that offers no benefit, especially since it was done bilaterally. In addition, it was crucial to evaluate the block’s dermatomal level prior to induction of anesthesia to verify its success and permit the patients from the two interventional groups to participate in the trial. However, by adhering to standardized protocol, we attempted to minimize any potential bias, and the surgeons, statisticians, anesthesiologists, and data collectors were all blind to the group assignments.

While the findings of this study are promising, several limitations are worth noting. First, the study was conducted in a single center, which may limit the generalizability of the results. Second, the allocation process was unblinded to patients which could introduce performance bias. Third, the study did not account for potential confounders such as individual patient comorbidities that could influence outcomes. Additionally, complications such as hypotension need to be scrutinized more deeply to rule out other interfering factors to ascertain their incidence with bilateral ESP. Finally, the single shot of the RIB and ESP blocks and the short follow-up period precluded the detection of blocks’ complications and long-term benefits for chronic pain. Therefore, future studies with larger sample sizes and longer follow-up periods are needed to validate these findings.

## Conclusion

Ultrasound-guided RIB is more effective than ESP in managing pain after reduction mammoplasty. It prolongs the duration of analgesia, reduces postoperative nalbuphine consumption and is associated with a lower incidence of complications; hence, RIB can be implemented as a promising alternative in bilateral breast surgeries.

## Data Availability

The corresponding author can provide the data utilized and analyzed in this study upon reasonable request.

## Abbreviations

ESP: Erector spinae plane block
LA: Local anesthetics
LAST: Local anesthetic systemic toxicity
MRM: Modified radical mastectomy
NRS: Numerical rating scale
PACU: Postanesthesia care unit
PCA: Patient-controlled analgesia
PECS-II: Pectoral nerve block II
PONV: Postoperative nausea and vomiting
PVB: Paravertebral block
RIB: Rhomboid intercostal block
SAP: Serratus anterior plane block
VAS: Visual analogue score scale
VATS: Video-assisted thoracoscopic surgery
